# Supplementary material for: Nurse Cultural Competence-cultural adaptation and validation of the Polish version of the Nurse Cultural Competence Scale and preliminary research results
Source: PLoS One. 2020 Oct 16;15(10):e0240884. doi: 10.1371/journal.pone.0240884 (PMC7567385; doi:10.1371/journal.pone.0240884)
Supplement: S1 Checklist — (DOC) [file pone.0240884.s001.doc]

**STROBE Statement—Checklist of items that should be included in reports of *cross-sectional study***

|  | Item No | Recommendation | Comments | |
| --- | --- | --- | --- | --- |
| Title and abstract | | | | |
|  | 1 | (*a*) Indicate the study’s design with a commonly used term in the title or the abstract | **The title includes this information.** | |
|  |  | (*b*) Provide in the abstract an informative and balanced summary of what was done and what was found | **The abstract includes this information.** | |
| Introduction | | | | |
| Background/rationale | 2 | Explain the scientific background and rationale for the investigation being reported | **This information is provided throughout the Introduction.** | |
| Objectives | 3 | State specific objectives, including any prespecified hypotheses | **This information is provided in the final paragraph of the Introduction.** | |
| Methods | | | | |
| Study design | 4 | Present key elements of study design early in the paper | **This information is provided in the Methods, in the subsection on Procedure.** | |
| Setting | 5 | Describe the setting, locations, and relevant dates, including periods of recruitment, exposure, follow-up, and data collection | **This information is provided in the Methods, in the subsection of Participants and data collection.** | |
| Participants | 6 | Give the eligibility criteria, and the sources and methods of selection of participants | **This information is provided in the Methods, in the subsection of Participants and data collection** | |
| Variables | 7 | Clearly define all outcomes, exposures, predictors, potential confounders, and effect modifiers. Give diagnostic criteria, if applicable | **This information is provided in the Methods.** | |
| Data sources/ measurement | 8* | For each variable of interest, give sources of data and details of methods of assessment (measurement). Describe comparability of assessment methods if there is more than one group | **This information is provided in the Methods, under the subsections on Instruments.** | |
| Bias | 9 | Describe any efforts to address potential sources of bias | **This information is provided in the Methods section, under the subsection on Statistical Analyses.** | |
| Study size | 10 | Explain how the study size was arrived at | **This information is provided in the Methods.** | |
| Quantitative variables | 11 | Explain how quantitative variables were handled in the analyses. If applicable, describe which groupings were chosen and why | **This information is provided in the Methods.** | |
| Statistical methods | 12 | (*a*) Describe all statistical methods, including those used to control for confounding | **This information is provided in the Methods section, under the subsection of Statistical Analyses.** | |
|  |  | (*b*) Describe any methods used to examine subgroups and interactions | **This information is provided in the Methods section, under the subsection of Statistical Analyses.** | |
|  |  | (*c*) Explain how missing data were addressed | **This information is provided in the Results section, under the subsection of Characteristics of the participants.** | |
|  |  | (*d*) If applicable, describe analytical methods taking account of sampling strategy | **N/A** | |
|  |  | (*e*) Describe any sensitivity analyses | **This information is provided in the Methods section, under the subsection of Statistical Analyses.** | |
| Results | | | |  |
| Participants | 13* | (a) Report numbers of individuals at each stage of study—eg numbers potentially eligible, examined for eligibility, confirmed eligible, included in the study, completing follow-up, and analysed | **This information is provided in the Results section, under the subsection of Characteristics of the participants.** |  |
|  |  | (b) Give reasons for non-participation at each stage | **This information is provided in the Results section, under the subsection of Characteristics of the participants.** |  |
|  |  | (c) Consider use of a flow diagram | **N/A** |  |
| Descriptive data | 14* | (a) Give characteristics of study participants (eg demographic, clinical, social) and information on exposures and potential confounders | **This information is provided in the Results section, under the subsection Characteristics and in Table 1** |  |
|  |  | (b) Indicate number of participants with missing data for each variable of interest | **This information is provided in the Results section, under the subsection of Characteristics of the participants.** |  |
| Outcome data | 15* | Report numbers of outcome events or summary measures | **This information is provided in the Results section, under the subsection Assessment of the relevance of the factors of the Polish adaptation NCCS-P** |  |
| Main results | 16 | (*a*) Give unadjusted estimates and, if applicable, confounder-adjusted estimates and their precision (eg, 95% confidence interval). Make clear which confounders were adjusted for and why they were included | **This information is provided in the Results section** |  |
|  |  | (*b*) Report category boundaries when continuous variables were categorized | **This information is provided in the Results section** |  |
|  |  | (*c*) If relevant, consider translating estimates of relative risk into absolute risk for a meaningful time period | **N/A** |  |
| Other analyses | 17 | Report other analyses done—eg analyses of subgroups and interactions, and sensitivity analyses | **N/A** |  |
| Discussion | | | |  |
| Key results | 18 | Summarise key results with reference to study objectives | **This information is provided in the Discussion.** |  |
| Limitations | 19 | Discuss limitations of the study, taking into account sources of potential bias or imprecision. Discuss both direction and magnitude of any potential bias | **This information is provided in the Discussion section, under the subsection Limitations of the research project** |  |
| Interpretation | 20 | Give a cautious overall interpretation of results considering objectives, limitations, multiplicity of analyses, results from similar studies, and other relevant evidence | **This information is provided in the Discussion.** |  |
| Generalisability | 21 | Discuss the generalisability (external validity) of the study results | **This information is provided in the Discussion in subsection Implications for nursing and Clinical significance** |  |
| Other information | | |  |  |
| Funding | 22 | Give the source of funding and the role of the funders for the present study and, if applicable, for the original study on which the present article is based | **We have provided this information in the section titled “Funding”.** |  |
| Supporting Information | files | Please include captions for your Supporting Information files (STROBE_checklist_Article_NCCS.doc changed from "other" to "supporting information" item type | **Matrix model of results for Table 2.** |  |

*Give information separately for cases and controls in case-control studies and, if applicable, for exposed and unexposed groups in cohort and cross-sectional studies.
