# Supplementary material for: Nurse Cultural Competence-cultural adaptation and validation of the Polish version of the Nurse Cultural Competence Scale and preliminary research results
Source: PLoS One. 2020 Oct 16;15(10):e0240884. doi: 10.1371/journal.pone.0240884 (PMC7567385; doi:10.1371/journal.pone.0240884)
Supplement: S1 Table — (DOCX) [file pone.0240884.s002.docx]

**S1 Table. The model matrix^a^ for Table 2.**

| **Subscales / items** | Component | | | |
| --- | --- | --- | --- | --- |
|  | **NCCS-CS-P** | **NCCS-CA-P** | **NCCS-CSe-P** | **NCCS-CK-P** |
| Item 1 | ,146 | ,304 | ,026 | -,024 |
| Item 2 | ,197 | ,297 | -,443 | ,045 |
| Item 3 | ,065 | ,321 | -,420 | ,326 |
| Item 4 | ,047 | ,670 | ,016 | ,011 |
| Item 5 | -,195 | ,633 | -,013 | ,049 |
| Item 6 | ,012 | ,705 | ,094 | -,022 |
| Item 7 | ,024 | ,551 | ,138 | -,116 |
| Item 8 | ,021 | ,740 | ,064 | -,070 |
| Item 9 | ,009 | ,498 | ,007 | ,116 |
| Item 10 | -,119 | ,124 | -,230 | ,564 |
| Item 11 | -,060 | ,309 | ,055 | ,387 |
| Item 12 | -,001 | -,078 | ,137 | ,545 |
| Item 13 | ,124 | -,023 | ,003 | ,714 |
| Item 14 | ,122 | ,066 | ,063 | ,557 |
| Item 15 | -,010 | -,011 | ,040 | ,706 |
| Item 16 | ,073 | -,061 | ,117 | ,729 |
| Item 17 | ,149 | -,107 | ,145 | ,651 |
| Item 18 | ,239 | -,034 | -,015 | ,639 |
| Item 19 | ,196 | -,163 | ,185 | ,617 |
| Item 20 | ,258 | ,216 | ,480 | ,094 |
| Item 21 | -,018 | ,078 | ,447 | ,004 |
| Item 22 | -,018 | ,084 | ,703 | ,114 |
| Item 23 | ,045 | ,015 | ,279 | ,056 |
| Item 24 | -,052 | ,151 | ,409 | -,060 |
| Item 25 | ,186 | ,146 | ,457 | ,144 |
| Item 26 | ,086 | ,176 | ,534 | ,282 |
| Item 27 | -,016 | ,095 | ,548 | ,122 |
| Item 28 | ,252 | -,101 | ,472 | ,323 |
| Item 29 | ,311 | -,014 | ,524 | ,130 |
| Item 30 | ,364 | ,139 | ,384 | ,054 |
| Item 31 | ,419 | ,008 | ,379 | ,153 |
| Item 32 | ,429 | -,063 | ,363 | ,320 |
| Item 33 | ,434 | -,050 | ,324 | ,333 |
| Item 34 | ,624 | -,033 | ,209 | ,170 |
| Item 35 | ,585 | ,033 | ,177 | ,117 |
| Item 36 | ,338 | -,069 | ,434 | -,019 |
| Item 37 | ,931 | ,061 | -,044 | -,055 |
| Item 38 | ,942 | ,053 | -,152 | ,025 |
| Item 39 | ,943 | ,052 | -,052 | -,017 |
| Item 40 | ,896 | -,048 | -,059 | ,104 |
| Item 41 | ,881 | -,004 | -,105 | ,087 |

Legend: Method of extracting factors - main components. Rotation method - Oblimin with Kaiser normalization.

a. Rotation converged in 9 iterations.
