# Supplementary material for: Nurse Cultural Competence-cultural adaptation and validation of the Polish version of the Nurse Cultural Competence Scale and preliminary research results
Source: PLoS One. 2020 Oct 16;15(10):e0240884. doi: 10.1371/journal.pone.0240884 (PMC7567385; doi:10.1371/journal.pone.0240884)
Supplement: Attachment — Submitted filename: Response to Reviewers.docx [file pone.0240884.s003.docx]

**Respected Reviewers,**

September 4, 2020

We wish to submit a revision of the manuscript “Nurse Cultural Competence-cultural adaptation and validation of the Polish version of the Nurse Cultural Competence Scale and preliminary research results” (Manuscript ID: PONE-D-20-07812) for further consideration by the PLOS ONE.

We do confirm that we have taken all the valuable reviewers’ comments under consideration and applied changes to the manuscript. The improvements are marked in blue in the manuscript.

|  | **Response** | |
| --- | --- | --- |
| **EDITORIAL CORRECTIONS REQUIRED** | | |
| Please include the following items when submitting your revised manuscript:   - A rebuttal letter that responds to each point raised by the academic editor and reviewer(s). You should upload this letter as a separate file labeled 'Response to Reviewers'. - A marked-up copy of your manuscript that highlights changes made to the original version. You should upload this as a separate file labeled 'Revised Manuscript with Track Changes'. - An unmarked version of your revised paper without tracked changes. You should upload this as a separate file labeled 'Manuscript'. | | **Dear Editors,**  Thank you for your input on how we can improve our [manuscript.as](http://manuscript.as/) well as instructions as to how to meet all necessary requirements. |
| 1.Please ensurethatyour manuscript meets PLOS ONE's style requirements, including those for file naming. The PLOS ONE style templates can be found at[https://journals.plos.org/plosone/s/file?id=wjVg/PLOSOne_formatting_sample_main_body.pdf](about:blank) and  https://journals.plos.org/plosone/s/file?id=ba62/PLOSOne_formatting_sample_title_authors_affiliations.pdf | | **Dear Editors,**  Thank you very much for reminding us of the principles of preparing the manuscript for printing. All guidelines have been met. The manuscript language style has againbeen reviewed by a native speaker. |
| 2. Please clarify in your Methods section whether the questionnaire is published under a CC-BY license, or whether you obtained permission from the publisher to reproduce the questionnaire in this manuscript. Please explain any copyright or restrictions on this questionnaire. | | **Dear Editors,**  we have made changes to the text of the article, after indicating the source: *Perng SJ, Watson R. Construct validation of the Nurse Cultural Competence Scale: a hierarchy of abilities. J Clin Nurs. 2012;21(11-12): 1678-1684. doi: 10.1111/j.1365-2702.2011.03933.x* and we have removed the quoted passages.  We obtained the authors' consent to use and publish the scale, but despite our best efforts, we were unable to obtain formal consent for the publication of the fragment of the text by Wiley Publishing. |
| 3. Please include your tables as part of your main manuscript and remove the individual files. Please note that supplementary tables (should remain/ be uploaded) as separate "supporting information" files. | | **Dear Editors,**  “Supporting information” files have been appended to the manuscript containing the model matrix outcome for Table 2. |
| 4. Please include captions for your Supporting Information files (STROBE_checklist_Article_NCCS.doc changed from "other" to "supporting information" item type) at the end of your manuscript, and update any in-text citations to match accordingly. Please see our Supporting Information guidelines for more information: http://journals.plos.org/plosone/s/supporting-information. | | **Dear Editors,**  Changes made to the text of the manuscript and the relevant source literature as well as the entry in STROBE have been revised in accordance with these guidelines. |
| **COMMENTS FROM THE REVIEWER 1** | | |
| . I only have a few suggestions: - the abstract should report the factor structure | | **Dear Reviewer,**  Thank you very much for your opinion and all of your suggestions for making our manuscript better.  This extract has been added in the abstract: page 1-2  The Cronbach's alpha reliability coefficient for the NCCS-P scale was 0.94, with the subscale values ranging from 0.72 to 0.95. Factor validity analysis of the Polish adaptation of the NCCS-P scale pointed to its four-factor structure. The Kaiser-Mayer-Olkin sampling adequacy test was 0.905, and the Bartlett test of sphericity result was χ2 = 5755.107; df = 820; p < 0.001. The four-factor structure is affirmed by the Kaiser criterion and the scree test result. |
| - for the translation procedure it might be good to give a reference to a standard procedure eg. the WHO procedure (If you used it: [https://www.who.int/substance_abuse/research_tools/translation/en/](about:blank)) or whatever procedure and to say how closely you followed this. | | **This content has been added on page 10:** As part of the adaptation procedure of the original version, the NCCS was translated into Polish by two independent translators. Next, a panel of experts made up of nurses of varying professional experience and working in various positions, developed version 1.0 of the NCCS scale, which was re-translated into English by an independent translator who was a native speaker of English. After a comparison of the original and adapted 1.0 versions, a pilot study was conducted with 15 nurses. The interviewer, who was one of the authors of the document, held discussions with the pilot study participants on the clarity and comprehensibility of the survey items, which made it possible to form the final version of the NCCS, which was subjected to the further examinations presented in this article. Every stage of the language adaptation of the scale has been documented. |
| 2. in Table 2 please present all the loadings on all the factors and not only those on putative factors | | The model matrix for Table 2 has been included in the supplementary materials. |
| 3. minor points: Registered Nurse should have capitals; data are plural | | Thank you for your observations. These corrections have been made. |
| 4. finally in limitations maybe mention that an additional and now more conventional method estimating the number of factors to be rotated - Monte Carlo Parallel analysis - could have been used and, if you have not already said it then the structure could be studied using confirmatory factor analysis in future. | | In this paper, Exploratory Factor Analysis was conducted. The structure of data could be studied using confirmatory factor analysis (CFA) in future analyze. CFA will allow for testing the number of factors (latent constructs) that best fit the model being examined. |
| **COMMENTS FROM THE REVIEWER 2** | | |
| Below are just a few comments. 1. The author(s) use the term "cultural meetings" in reference to Campihna-Bacote's model and tool. However, the term is cultural encounter. Conceptually, meetings and encounter can be defined differently. | | **Dear Reviewer,**  Thank you very much for your opinion and all of your suggestions for making our manuscript better. The changes have been made and are highlighted in the manuscript text. |
| 2. I would add some previous research findings to show validity and use of the tool. | | The following current research results have been added:  -M-H, Lin, H-C Hsu, Effects of a cultural competence education programme on clinical nurses: A randomised controlled trial, Nurse Education Today,Volume 88, 2020, 104385, ISSN 0260-6917, [https://doi.org/10.1016/j.nedt.2020.104385](about:blank)].  -Terzoni S, Ferrara P, Sala E, Destrebecq A, Trombetta N. La versioneitalianadella Nurse Cultural Competence Scale: validazionesuglistudenti di Infermieristica [The Italian version of the Nurse Cultural Competence Scale: validation on a sample of Nursing students]. Prof Inferm. 2020;73(1):13-20. doi:10.7429/pi.2020.721011 |
| 3. There are some grammatical and flow issues within the article | | The translator, who is a native speaker of English, has made a fresh revision of the manuscript. |
| 4. The statistics are sound and are congruent with other research findings in this area. I am not well-versed on how well studied this topic is in Poland. I am not sure what new findings this adds to the literature | | In the Polish literature there is a palpable lack of tools for assessing cultural competences of nurses as significant providers of health services and caregivers. The Polish version of the NCCS will make it possible to assess the current state of the cultural competences of nurses as well as to monitor their development in a reasonable way. |
